# On-site fabrication of Bi-layered adhesive mesenchymal stromal cell-dressings for the treatment of heart failure

**DOI:** 10.1016/j.biomaterials.2019.04.014

**Published:** 2019-07

**Authors:** Kazuya Kobayashi, Yuki Ichihara, Nobuhiko Sato, Nobuyoshi Umeda, Laura Fields, Masafumi Fukumitsu, Yoshiyuki Tago, Tomoya Ito, Satoshi Kainuma, Mihai Podaru, Fiona Lewis-McDougall, Kenichi Yamahara, Rakesh Uppal, Ken Suzuki

**Affiliations:** a William Harvey Research Institute, Barts and the London School of Medicine, Queen Mary University of London, United Kingdom; b Kaneka Corporation, Osaka, Japan; c Transfusion Medicine and Cellular Therapy, Hyogo College of Medicine, Japan

**Keywords:** Mesenchymal stromal cell, Myocardial repair, Bioengineering, Cardiac patch, Fibrin sealant film

## Abstract

Mesenchymal stromal/stem cell (MSC)-based therapy is a promising approach for the treatment of heart failure. However, current MSC-delivery methods result in poor donor cell engraftment, limiting the therapeutic efficacy. To address this issue, we introduce here a novel technique, epicardial placement of bi-layered, adhesive dressings incorporating MSCs (MSC-dressing), which can be easily fabricated from a fibrin sealant film and MSC suspension at the site of treatment. The inner layer of the MSC dressing, an MSC-fibrin complex, promptly and firmly adheres to the heart surface without sutures or extra glues. We revealed that fibrin improves the potential of integrated MSCs through amplifying their tissue-repair abilities and activating the Akt/PI3K self-protection pathway. Outer collagen-sheets protect the MSC-fibrin complex from abrasion by surrounding tissues and also facilitates easy handling. As such, the MSC-dressing technique not only improves initial retention and subsequent maintenance of donor MSCs but also augment MSC's reparative functions. As a result, this technique results in enhanced cardiac function recovery with improved myocardial tissue repair in a rat ischemic cardiomyopathy model, compared to the current method. Dose-dependent therapeutic effects by this therapy is also exhibited. This user-friendly, highly-effective bioengineering technique will contribute to future success of MSC-based therapy.

## 1 Introduction

Mesenchymal stromal/stem cells (MSCs) have a potential to treat diseases that are difficult to cure with existing treatments, including severe heart failure [1,2]. Although MSCs rarely transdifferentiate to cardiomyocytes *in vivo*, these cells have a substantial capacity to induce myocardial tissue repair through their secretome (so-called paracrine effect) [[2], [3], [4], [5]]. In addition, MSCs have a potential for allogeneic donation [1,2], which would enable an off-the-shelf supply of donor cells. This is the key for cell-based therapies to become a widely-adopted standard treatment. Following a number of successful animal studies, clinical trials have proven the safety and feasibility of the MSC-based therapy; however, the therapeutic efficacy observed to date has not been as substantial as anticipated, albeit not negative, indicating that refinement of the protocol is required.

One of the most important issues associated with MSC-based therapy for heart failure is the sub-optimal cell-delivery route [2,6]. While intramyocardial (IM) and intracoronary injections have been commonly used in both pre-clinical and clinical studies, these methods are known to result in poor donor cell engraftment [2,[6], [7], [8], [9], [10]]. In addition, these methods carry a risk of complications, such as arrhythmia occurrence and coronary embolism [7,9,11,12]. Recently, epicardial placement, which is based upon cell-placement onto the heart surface with aid of bioengineering techniques (i.e. cardiac patches), has been proposed to be a more effective and safer cell-delivery method [2,6,[13], [14], [15], [16], [17], [18], [19], [20]]. A range of cardiac patch products have been reported in translational studies [[21], [22], [23]], among which skeletal myoblast-sheets [19] and pre-fabricated fibrin glue patch-incorporating cardiac progenitor cells [20] have already been applied to heart failure patients. However, these methods based on pre-fabricated bio-products are associated with limited availability due to highly-specialized cell culture steps in a clinical-grade cell processing facility, demanding quality control and complicated logistics. In addition, these products require sutures or additional glue to stabilize them on the heart surface. Suturing has a risk of myocardial injury, while glues may not be adhesive enough to fix a large cardiac patch on the beating heart surface. Also, glues may form an unwanted gap between the cardiac patch and epicardium, inhibiting the permeation of MSC's secretome into the myocardium, which will limit the paracrine effect.

Adhesive hydrogel-MSC complexes, which can be produced at the point of the treatment, may tackle such limitations [[15], [16], [17], [18]]. For example, PuraMatrix^®^ (3-D Matrix, Inc. Japan) is a self-assembling peptide hydrogel developed for hemostasis [24], and we have reported that epicardial placement of an instantly produced PuraMatrix^®^-MSC complex resulted in increased donor cell survival and enhanced therapeutic effects, compared to IM injection, in rat heart failure models [15]. We have also demonstrated that epicardial placement of a complex of MSCs and fibrin glue, which is currently used for hemostasis during surgery, improved donor cell engraftment and augmented therapeutic outcome, compared to IM injection, in a rodent model [16]. However, we recognized that placement of these gel-MSC mixtures onto the lateral wall of the beating hearts in either small rodents or pigs was often problematic due to positioning, which led to significant loss of the donor cells.

**Fig. 1:**
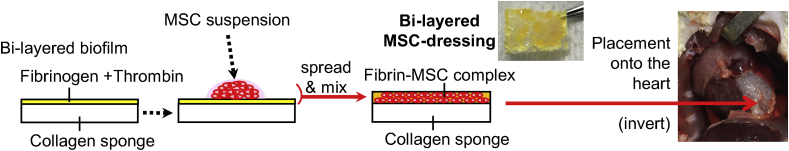
MSC-dressing technique. A schematic diagram describing the MSC-dressing technique for the treatment of heart failure.

We now consider that all above issues could be addressed by the use of a bi-layered, fibrin sealant film. A suitable example is TachoSil^Ⓡ^ (Takeda Pharmaceutical Company, Japan) [25], which consists of a collagen-sheet, one side of which is coated with fibrinogen and thrombin (Fig. 1). The collagen sheet is 0.5 cm in thickness composed of collagen layers having a hexagonal and honeycomb-like spatial structure [25,26]. Its degradation time is reported to be within 12 weeks when the dressing was placed onto the rat pleura [27]; however, this is subject to the surrounding circumstances [28]. Spreading of the MSC suspension on the fibrinogen/thrombin-coated side would facilitate formation of a fibrin gel that incorporates MSCs. The resultant adhesive film containing the MSC-fibrin complex (bi-layered MSC-dressing) could be directly placed onto any part of the heart surface, which would quickly and firmly adhere due to the fibrin's adhesive property without suture or extra glues. In addition, the fibrin gel would retain donor MSCs within, offering a scaffold suitable for their survival and function. On the other hand, the outer collagen sheet of the MSC-dressing would prevent the MSC-fibrin complex from external damage, including mechanical abrasion by surrounding tissues, while making this product easy to handle. Collectively, it was hypothesized that this user-friendly MSC-dressing technique would enhance donor MSC engraftment and improve the therapeutic efficacy of MSC-based therapy for heart failure.

## 2 Materials and methods

All studies were performed with the approval of the institutional ethics committee at Queen Mary University of London and the Home Office, UK. The animal investigations conform to the Principles of Laboratory Animal Care formulated by the National Society for Medical Research and the Guide for the Care and Use of Laboratory Animals (United States National Institutes of Health Publication, 1996). All *in vivo* and *in vitro* procedures and assessments were carried out in a blinded manner where possible.

### 2.1 Isolation of MSCs

#### 2.1.1 Human amnion-derived MSCs

The fetal appendage (amnion) was aseptically collected from pregnant patients with informed consent and placed in a sterile vat containing physiological saline solution. Amnion was washed with Ca/Mg-free Hanks' balanced salt solution (HBSS) to remove the blood and clots. 240 PU/mL collagenase and 200 PU/mL dispase I were added to the amnion, and stirred for 90 min at 37 °C. The resulting cell suspension containing amnion-derived MSC was filtered with nylon mesh filter, while remaining undigested amnion was removed. Collected amnion-derived MSCs were cultured in Cell Stack (6000 cells/cm^2^) with αMEM (Gibco) containing 10% FBS (Sigma-Aldrich), l-glutamine (200 mM; Gibco), penicillin (100 U/ml) and streptomycin (100 μg/ml; Sigma-Aldrich), at 37 °C in a humidified atmosphere containing 95% air and 5% CO_2_. Cells at passage 4 or 5 were used for studies.

#### 2.1.2 Rat amnion-derived MSCs

Amnion-derived MSCs were collected from the fetal membrane of pregnant Lewis rats (pregnant day 19–20; Charles River, UK) and expanded following a reported protocol [29]. Collected cells were placed in 25 cm^2^ flasks (Nunc) with an initial plating concentration of approximately 1 × 10^6^ cells/cm^2^ and cultured in αMEM with 10% inactivated FBS containing l-glutamine (200 mM), penicillin (100 U/ml) and streptomycin (100 μg/ml), at 37 °C in a humidified atmosphere containing 95% air and 5% CO_2_. Cells at passage 4 or 5 were used for studies.

#### 2.1.3 Rat bone marrow-derived MSCs

Rat bone marrow-derived MSCs were collected from the bone marrow of the tibias and femurs of male Lewis rats (100–150 g; Charles River UK) and expanded as we have described previously [13,30]. Collected cells were cultured in αMEM with 20% inactivated FBS containing l-glutamine (200 mM), penicillin (100 U/ml) and streptomycin (100 μg/ml) at 37 °C in a humidified atmosphere containing 95% air and 5% CO_2_. Cells at passage 4 or 5 were used for studies.

### 2.2 Characterization of MSCs

#### 2.2.1 Cell surface marker detection by flow-cytometric analysis

Rat MSCs were stained with 1:100 dilution of fluorescein isothiocyanate-conjugated anti-rat CD34 (Santa Cruz Biotechnology, USA), CD45 (Chemicon; Hampshire, UK), CD90 (Abcam, Cambridge, UK) or Alexa 647-conjugated anti-rat CD29 (Biolegend, London, UK) antibodies. For human MSCs, anti-human CD34, CD45, CD73 (BD Biosciences, USA) or PE-conjugated anti-human CD29 (Biolegend) antibodies were used. Corresponding isotype-matched control antibodies were used for negative controls. All antibodies were used at 1:100 dilution following instructions stipulated by the company's guidelines. Samples were analyzed using the LSRFortessa (BD Biosciences, USA).

#### 2.2.2 Osteogenic and adipogenic differentiation assay

Cultured MSCs were subjected to adipogenic or osteogenic differentiation medium. Adipogenic differentiation medium was α-MEM supplemented with 100 μM isobutyl methylxanthine (Sigma-Aldrich, UK), 60 μM indomethacin (Fluka; Dorset, UK), 1 μg/ml insulin (Sigma-Aldrich), and 0.5 μM hydrocortisone (Sigma-Aldrich), while osteogenic differentiation medium was α-MEM supplemented with 0.1 μM dexamethasone (Sigma-Aldrich), 10 mM β-glycerophosphate (Sigma-Aldrich), and 0.05 mM ascorbic acid (Sigma-Aldrich). After 3 weeks of incubation, cells were fixed with 4% paraformaldehyde and stained with Oil red O (Fluka) for detecting adipocytes containing lipid vacuoles or with Alizarin red (Fluka) to detect osteocytes containing calcium deposits.

### 2.3 Maintenance, expansion and CM-DiI labelling of MSCs

The culture medium was aspirated and changed every 48–72 h without additional washing. When cell confluency reached 80–90%, cells were passaged by detachment using 0.25% Trypsin/0.2% EDTA (Sigma). Plating concentrations for subsequent passages were 6000 cells/cm^2^ (human amnion-derived MSCs) and 1 × 10^4^ cells/cm^2^ (rat amnion-derived and bone marrow-derived MSCs). When required, MSCs were cryopreserved in a mixed CP-1 solution (a saline containing 5 wt% DMSO, 6 wt% hydroxyethyl starch and 4% human serum albumin) diluted with 50 wt% PRMI1640 at a density of 8 × 10^7^ cells/ml. MSCs were used for experiments before 6 passages. For histological tracking of donor cells, MSCs were labeled with CM-DiI (Molecular Probes, UK) as previously described [[13], [14], [15], [16],^31^].

### 2.4 Optimization of the protocol to fabricate an adhesive bi-layered MSC-dressing

The protocol to produce an MSC-dressing was optimized *in vitro* by mixing suspension of human amnion-derived MSCs with a fibrin sealant film, TachoSil^®^ (Takeda Pharmaceutical Company, Japan) [25]. Different volumes of the MSC suspension in HBSS including calcium and magnesium salt [HBSS (+); ThermoFisher Scientific, UK] were loaded onto the fibrinogen/thrombin side of the 1 × 1 cm film and spread over the surface using a pipette tip (Fig. 1). Formation of the MSC-fibrin complex and spillage of solution when inverted were visually assessed to select the most appropriate volume.

### 2.5 Assessment of MSC viability in the MSC-dressing

TachoSil^®^ (1 cm^2^) was loaded with a range of numbers of MSCs and placed in a 12-well plate (the fibrin layer upturning) with 200 ml αMEM (Gibco) containing 10% FBS l-glutamine (200 mM; Gibco), penicillin (100 U/ml) and streptomycin (100 mg/ml; Sigma), at 37 °C in a humidified atmosphere containing 95% air/5% CO_2_. At specific time points, the TachoSil^®^-MSC complex was collected into a 1.5 ml tube containing 500 μl saline added with 10 IU/ml Nattokinase (NSK-SD, Japan Bio Science Laboratory) [32]. After 10-min incubation at 37 °C, the majority of MSCs were dissociated, and the remnant TachoSil^®^ was removed. Remaining cell suspension was added with 500 μl αMEM and centrifuged at 400 g for 3 min. Pelleted MSCs were resolved in 100 μl PBS and stained for 7-AAD (Via Probe™, BD Bioscience) for 15 min 7-ADD negative ratios detected using flow cytometer were calculated as the MSC viability.

### 2.6 MSC-dressing therapy in a rat ischemic cardiomyopathy model

MI was induced in female Lewis rats (175–200 g) by ligating the left coronary artery through left thoracotomy under isoflurane anesthesia and mechanical ventilation as described previously [[13], [14], [15], [16],^31^]. Successful MI induction was confirmed based on change in ventricular color and cardiac movement. Then the chest and skin were closed so that the animals were allowed to regain consciousness. At 4 weeks, rats were subjected to echocardiography, and those showing inappropriate left ventricular ejection fraction (LVEF >45% or <25%) were excluded from the study [14,31]. Included animals were randomly allocated into 6 treatment groups as described below. It was confirmed that pre-treatment cardiac function and diameter (4 weeks after MI induction) was equivalent among groups (Supplementary Table 1).

The heart was again exposed through re-left thoracotomy under mechanical ventilation. The epicardial heart surface was exposed, and 1 cm^2^ TachoSil^®^ loaded with 4 × 10^6^, 2 × 10^6^ or 1 × 10^6^ rat amnion-derived MSCs (MSC-dressing) was placed onto the heart surface to cover ischemic areas (DR-4m, DR-2m, and DR-1m groups, respectively). In addition, 4 × 10^6^ of rat amnion-derived MSCs were suspended in 200 μl HBSS (+) and intramyocardially injected into two sites (100 μl each), targeting the border and infarct areas (IM group) as we have described [13,31]. The Sham group received an open/close chest procedure only at 4 weeks after coronary artery ligation, whilst the DR-0 group were placed with TachoSil^®^ only (no MSC included) on the heart surface. After each treatment, the chest and skin were closed with sutures. Animals were allowed to fully recover and were returned to their cages.

### 2.7 Assessment of cardiac function

Transthoracic echocardiography was performed assessed pre-myocardial infarction (base-line) and at day 28 post-treatment by the Vevo-770 echocardiography machine (VisualSonics, Fujifilm, Amsterdam, Netherlands) under isoflurane inhalation administered *via* a nose cone as we have previously described [[14], [15], [16],^31^]. LV end-diastolic (LVEDD) and end-systolic (LVESD) dimensions, under stable, consistent heart rate, were measured using M mode. Left ventricular ejection fraction (LVEF) was calculated from the data obtained with 2-dimensional tracing. All data were collected blind from at least 3–5 different measurements.

Hemodynamic parameters were measured by using cardiac catheterization (SPR-320 and PVAN3.2; Millar Instruments, USA) as we have previously described [[14], [15], [16],^31^]. Briefly, under general anesthesia using isoflurane inhalation with a nose cone, the catheter was inserted into the left ventricular cavity through the right common carotid artery. Intra-LV pressure signals were measured with a transducer and conditioner (MPVS-300; Millar Instruments) and digitally recorded with a data acquisition system (PowerLab 8/30; AD Instruments, Oxford, UK). All data were collected from at least 5 different measurements in a blinded manner.

### 2.8 Histological studies

Immediately after cervical dislocation, the aorta of the rat was clamped under thoracotomy and ice-cold PBS with 16 mEq/l potassium was injected into the LV cavity using a 23G needle until the heart stopped beating. The heart was then perfused by injecting ice-cold 4% of paraformaldehyde in PBS into the LV cavity. It was cut into three pieces and embedded in OCT compound (VWR International) and frozen in isopentane chilled in liquid nitrogen. Cryosections (6–7 μm thick) were cut and incubated in PBS containing 0.1% of Triton X (Sigma-Aldrich) for 5 min at the room temperature, and the non-specific antibody binding sites were pre-blocked with the blocking buffer (PBS plus 5% of goat serum). Samples were then incubated with the following primary antibodies; monoclonal alpha sarcomeric actinin antibody [EA-53] (1:100 dilution; ThermoFisher), polyclonal anti-cleaved caspase-3 antibody (1:250, Cell Signaling), monoclonal anti-Ki67 antibody (1:50, Dako), and polyclonal anti-wheat germ agglutinin antibody (1:100; ThermoFisher) at 4 °C overnight. For isolectin B4 staining, biotinylated Griffonia simplicifolia lectin I-isolectin B4 (1:100 dilution Vector L-1104) was used. After rinsing in PBS 3 times for 5 min, the sections were next incubated with the fluorophore-conjugated secondary antibody (AlexaFluor 488-conjugated antibody; 1:300 dilution Invitrogen A-11001) together with 4′,6-diamidino-2-phenylindole (DAPI) for nuclear counterstaining, in the blocking buffer for 1 h at the room temperature. Stained sections were mounted with DAKO Fluorescence Mounting Medium and the digital images were acquired with an All-in-One microscope (Keyence, UK). For semi-quantitative assessments, eight different fields of the border areas (viable areas surrounding the infarct) per heart were randomly selected and assessed. To evaluate cardiomyocyte size, the cross-sectional area of transversely cut cardiomyocytes (having central nuclei and surrounded by circle-shaped capillaries) was measured for 50 cardiomyocytes per area. For CM-DiI-positive area measurement, the largest CM-DiI-stained slice among consecutive 60 slices at the mid-level of left ventricle was scanned and the CM-DiI-positive area was assessed by using ImageJ analysis software (National Institutes of Health).

In addition, 6–7-μm frozen sections were incubated in 1.5% of phosphomolybdic acid for 60 min, next in 0.1% of Picrosirius red for 15 min, and then in 0.5% of acetic acid solution for 3 min. After dehydration through increasing concentrations of ethanol to xylene, the sections were mounted using the DPX mounting medium (VWR International). The infarct size (the ratio of scar length to total left ventricular circumference) and the wall thickness were measured at 5 independent regions of the infarct area as previously described [13]. The quantity of the collagen volume fraction was calculated from 6 fields of each area per rat by using a National Institute of Health image-analysis software (ImageJ).

### 2.9 RT-PCR

To assess gene expression, total RNA was extracted from cells or LV walls using the Gene Jet PCR purification Kit (Thermo Scientific) and quantified with a Nano-Drop 8000 spectrophotometer (Thermo Scientific). cDNA was synthesized from 25 ng to 150 ng of total RNAs from the rat heart tissues and rat MSCs, respectively, by using the high-capacity cDNA Reverse Transcription Kit (Applied Biosystems). Real-time PCR was performed by a 7900HT (Applied Biosystems) with a SYBR Green I master mix (Roche) in following conditions: 95 °C for 10 min followed by 50 cycles at 95 °C for 15 s, 64 °C for 30 s and 72 °C for 30 s. TaqMan primers and probes were purchased from Applied Biosystems. Expression was normalized to ubiquitin C. Relative expression to the Sham group is presented in the figures.

### 2.10 MSC transplantation to quantitatively evaluate the donor cell retention in rat

Female Lewis rats (175–200 g; Charles River UK) were subjected to exposure of the heart and left coronary artery ligation through left thoracotomy and pericardiotomy under isoflurane anesthesia and mechanical ventilation as described previously [[13], [14], [15], [16],^31^]. For MSC-dressing therapy, 30 μl HBSS (+) containing 4 × 10^6^ male Lewis rat bone marrow-derived MSCs were loaded onto the fibrinogen/thrombin side of the 1 × 1 cm film and spread over the surface using a pipette tip. The freshly-produced MSC-dressing was placed on to the surface of the beating rat heart so that the MSC-fibrin complex contacted to the heart (Fig. 1). The majority of left ventricular free walls were covered by the MSC-dressing. For IM injection, 4 × 10^6^ of male, rat bone marrow-derived MSCs were suspended in 200 μl HBSS (+) and directly injected into two sites of the left ventricular free wall (100 μl each) [13,31]. After the treatment, the chest and skin were closed and the rats were allowed to recover under a heat source and were returned to their cages.

### 2.11 Quantitative assessment of donor cell presence

To quantitatively evaluate the presence of transplanted male donor cells in the female host heart, the Y chromosome specific *Sry* gene was assessed by real-time PCR (Prism 7900HT; Applied Biosystems, UK) [[14], [15], [16],^31^]. At specific time points after transplantation of male rat bone marrow-derived MSCs by the MSC-Dressing technique to the female rat heart, the whole ventricular walls were collected. Genomic DNA was extracted using the DNeasy Blood&Tissue kit (Qiagen), and *Sry* analysis was performed in technical duplicate. The signal in each sample was normalized to the amount of DNA by measuring the autosomal single-copy gene *Osteopontin* as an internal standard [[14], [15], [16],^31^]. To generate a standard curve, the ventricular walls from female rats were mixed with known numbers (1 × 10^7^, 1 × 10^6^, 1 × 10^5^, 1 × 10^4^) of male MSCs (*n* = 3).

### 2.12 In vitro culture of MSCs in fibrin

A total of 5 × 10^4^ rat amnion-derived MSCs were suspended in 50 μl of fibrinogen solution (from Beriplast P Combi-set, CSL Behring). This suspension was then placed in a single well of a 24-well plate and 50 μl of thrombin was then rapidly added to the well and mixed using the pipette tip. This formed the FG-MSC complex, which added with 100 μl of growth medium (αMEM with 20% inactivated FBS containing l-glutamine, penicillin and streptomycin). For the Control group, 5 × 10^4^ rat amnion-derived MSCs were plated on a 24-well plate with 200 μl growth medium. These were incubated at 37 °C and 5% CO_2_. At specific time points, cells were dissociated by adding 500 fibrin-degradation units of nattokinase dissolved in 100 μl of αMEM. This detached the MSCs from the well. Viability and number of collected MSCs were assessed using Countess Cell Counter (Invitrogen).

### 2.13 Assessment of tolerance of MSCs against H_2_O_2_*in vitro*

At Day 3 of the culture in either fibrin gel or ordinary culture medium, 100 μM of H_2_O_2_ was added to the rat amnion-derived MSC culture. At 1 h, supernatant was collected to detect LDH leakage using Thermo Scientific Pierce LDH Cytotoxicity Assay Kit. In addition, MSCs were collected as above and subjected to measurement of cell viability using Countess Cell Counter (Invitrogen).

### 2.14 Western blot

MSCs cultured for 3 days in either fibrin gel or ordinary culture medium were lysed in lysis buffer (0.15 M NaCl, 1 mM EDTA, 20 mM Tris pH 7.4, 10% glycerol, 1% Nonidet P-40, 10 mM NaF, 2 mM sodium orthovanadate, and protease inhibitor cocktail; Sigma) for 10 min on ice. Then, MSCs were collected with scalpel and all lysate were collected. These were then centrifuged at 20,000 RCF and supernatant were collected. After measuring the protein concentration (BioRad DC Protein Assay), 50–100 μg protein was mixed with NuPAGE LDS Sample Buffer (Thermo Fisher Scientific) and dithiothereitol (20 mM final concentration), and subjected to SDS-PAGE and Western blotting using anti-phosphorylated Akt (p-Akt; Ser 473 Cell Signaling, Cat 4051) or anti-phosphorylated PI3Kinase p85 alpha (phospho Y607) antibody (p-PI3K; ab182651, Abcam Biotechnology). Then, the labeled membrane was stripped, and re-probed with anti-Akt (Cell signaling, Cat 9272) or anti-PI3K p85 alpha antibody [M253] (ab86714, Abcam Biotechnology). Blots were scanned and semi-quantitative analysis was performed using ImageJ software. The proportion of the phosphorylated protein to the total protein was normalized to that of the corresponding Control group.

### 2.15 Statistical analysis

All values are expressed as mean ± SEM. Statistical comparison of two groups was performed using the Student's unpaired *t*-test. Other data were statistically analyzed with appropriate ANOVA (see each Figure Legend for details) followed by the Least Significant Difference test to compare groups. A value of p < 0.05 was considered statistically significant.

## 3 Results

### 3.1 Optimization of the protocol to fabricate an MSC-dressing

First, we optimized the technical protocol to produce a bi-layered adhesive MSC-dressing using human amnion-derived MSCs (see Supplementary Fig. 1 for their characters). Amnion-derived MSCs were primarily tested in this study, as this MSC type has a potential advantage in mass production due to their large initial yield, high proliferative capacity and less senescence [[33], [34], [35]]. As a film material, we chose a commercially available fibrin sealant film, TachoSil^®^, which is widely used for tissue sealing and hemostasis [25]. As a result of loading and spreading of different volumes of MSC suspension onto the fibrinogen/thrombin side of the film (Fig. 1), we identified that 25–40 μl of cell suspension per 1 cm^2^ film is the optimal volume range for the fabrication of an MSC-dressing (Supplementary Fig. 2a). Less than 25 μl suspension could not produce homogenous fibrin, while more than 40 μl suspension resulted in overflow. In terms of the MSC loading dose, we identified that an MSC suspension having a greater than 1.3 × 10^8^ cells/ml cell dose (namely 4 × 10^6^ MSCs on 1 cm^2^ film) became too viscous to handle using a pipette. We also confirmed that, when 30 μl of lower doses than 1.3 × 10^8^ cells/ml MSCs were loaded, the MSC viability was >90% for at least 6 h (Supplementary Fig. 2b).

### 3.2 Cardiac function improvement by MSC-dressing technique in a rat heart failure model

Using the above optimized protocol, we fabricated the MSC-dressings and examined the therapeutic efficacy of epicardial placement of these in a clinically-relevant post-MI ischemic cardiomyopathy model in rat. At 4 weeks after induction of MI, 1 cm^2^ MSC-dressings containing different numbers (0–4 × 10^6^) of rat amnion-derived MSCs (See Supplementary Fig. 3 for their characters) were produced and immediately placed onto the epicardial surface of a beating rat heart, targeting the per-infarct and infarct areas, under re-thoracotomy (Fig. 1; MSC-dressing technique). The MSC-dressing quickly and firmly adhered to the heart surface of both anterior and lateral ventricular walls. No leakage of MSC suspension occurred. No suture or glue was necessary to fix the MSC-dressing.

**Fig. 2:**
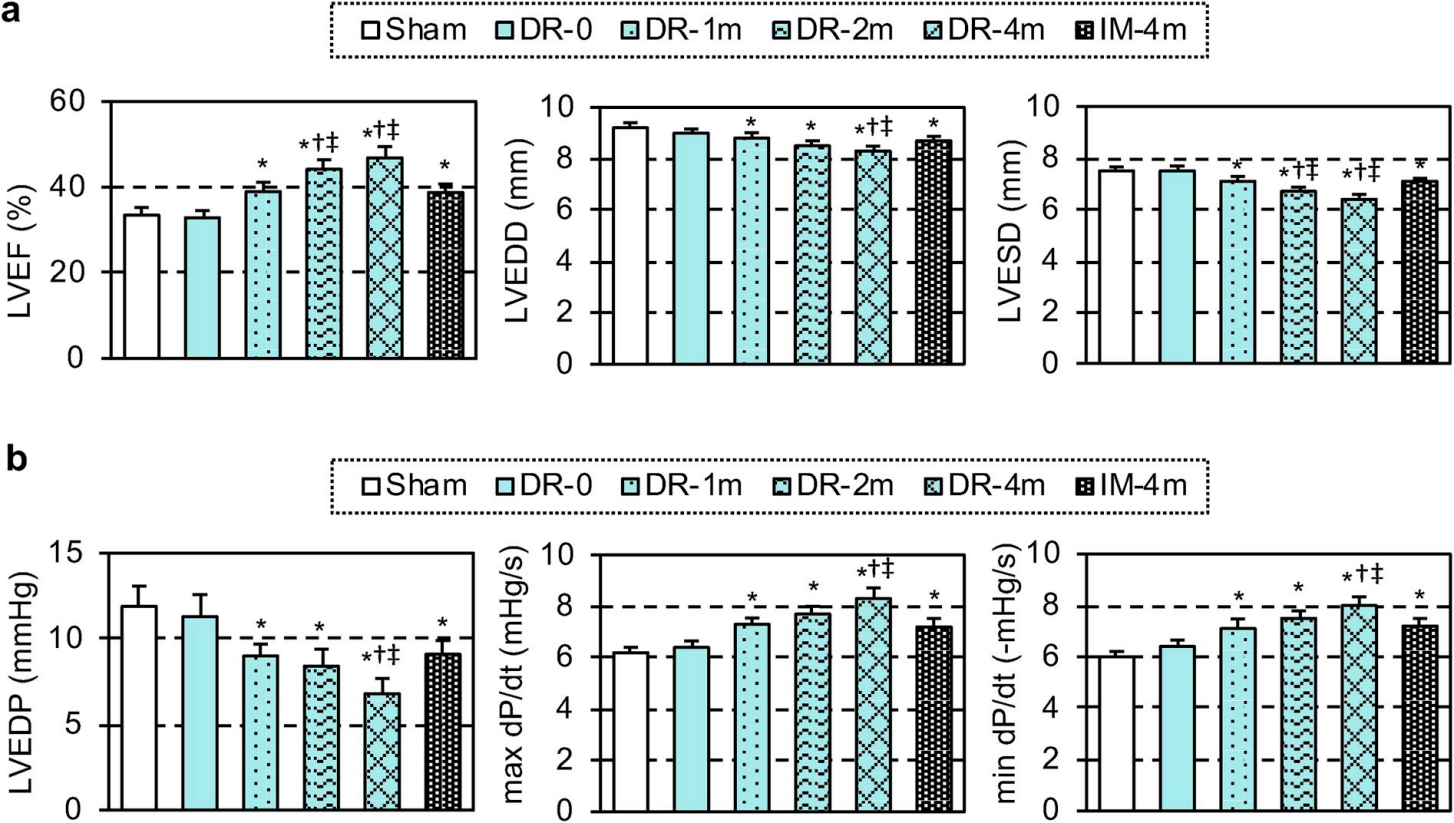
Enhanced cardiac function by MSC-dressing therapy in a rat heart failure model. Four weeks after left coronary artery ligation in rats, MSC-dressings (containing 1, 2 or 4** × **10^6^ rat amnion-derived MSCs; DR-1m, DR-2m, DR-4m groups, respectively), fibrin sealant film only (DR-0 group), or no treatment (Sham group) were placed onto the heart surface. Additional animals received IM injection of 4** × **10^6^ MSCs (IM-4m group). At 28 days post-treatment, cardiac function and structure measured by using echocardiography (**a**) and catheterization (**b**). LVEF, left ventricular ejection fraction; LVEDD, left ventricular end-diastolic dimension; LVESD, left ventricular end-systolic dimension; LVEDP, left ventricular end-diastolic pressure, n = 9–10 in each group; *p < 0.05 vs. Sham and DR-0 groups, ^†^p < 0.05 vs. DR-1m group, ^‡^p < 0.05 vs. IM-4m group. One-way ANOVA.

At 28 days post-treatment, echocardiography demonstrated that the MSC-dressing therapy improved cardiac function and reduced left ventricular dilatation in proportion to the MSC number transplanted (1 × 10^6^–4 × 10^6^), compared to the sham control group (Fig. 2a). IM injection of 4 × 10^6^ MSCs improved cardiac function, but this was less extensive compared to the dressing technique. Placement of the film alone (DR-0 group) did not change any cardiac parameters. In addition, cardiac catheterization showed that both systolic and diastolic cardiac performance was improved by the MSC-dressing therapy and IM injection compared to the sham control group, but with the effect being more extensive when using the dressing therapy (Fig. 2b).

### 3.3 Improved donor cell engraftment by MSC-dressing technique

**Fig. 3:**
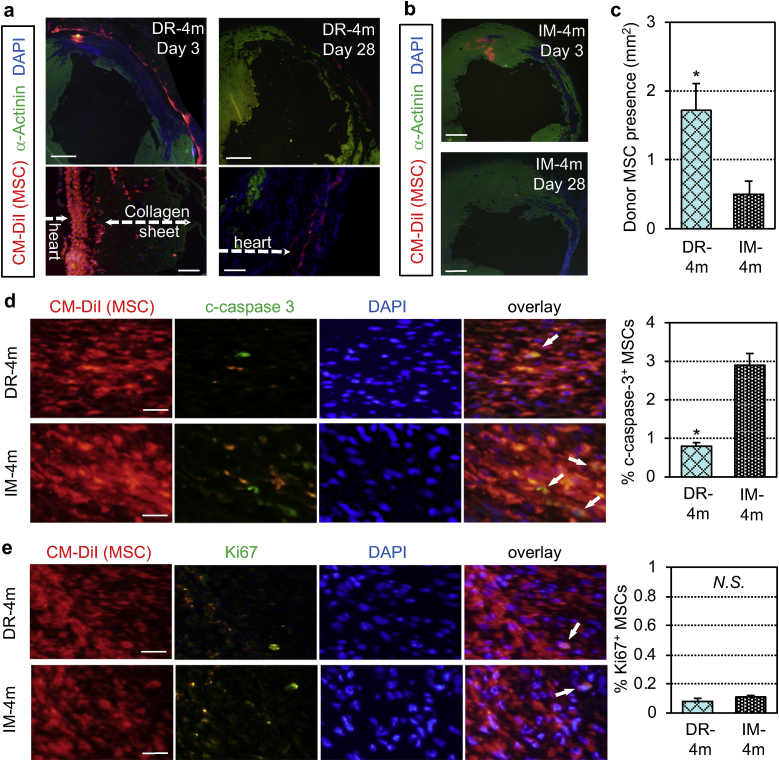
Donor cell behaviors after MSC-dressing therapy. Heart samples after MSC transplantation in a rat post-MI ischemic heart failure model were collected and analyzed by histological assessment for donor cell behaviors. The DR-4m and IM-4m groups were transplanted with 4** × **10^6^ rat amnion-derived MSCs at 4 weeks after induction of MI by the MSC-dressing therapy or IM injection, respectively. The DR-0 group received transplantation of fibrin sealant film only, while the Sham group received no treatment. Donor MSCs were pre-labeled with CM-DiI (red). (**a**) Immunohistostaining identifying donor MSC survival in the DR-4m group. *Scale bars = *1 mm *(upper panel) and* 100 μm *(lower panel).* (**b**) Presence and distribution of donor MSCs in the IM-4m group detected by immunohistostaining. *Scale bar = *1 mm*.* (**c**) Donor MSC presence (Y-axis) representing the CM-DiI-positive area calculated from day 3 immunohistostaining images. n = 5 in each group, *p < 0.05, Student's t-test. (**d, e**) Immunostaining for cleaved caspase 3 (c-caspase 3; **d**) and Ki67 (**e**) showing apoptosis and proliferation of donor MSCs (white arrows) at day 3 post-treatment. % c-caspase 3^+^ MSCs and Ki67^+^ MSCs (Y-axis) represent the ratio of the c-caspase 3^+^ and Ki67^+^ cells among the CM-DiI^+^ cells. *Scale bars = *50 μm, n = 5 in each group, *p < 0.05, N.S. = not significant, Student's t-test. . (For interpretation of the references to color in this figure legend, the reader is referred to the Web version of this article.)

Macroscopically, persistent adherence of the MSC-dressing to the heart at both anterior and lateral left ventricular walls was confirmed at days 1 and 3 post-transplantation. However, the structure of engrafted MSC-dressings was mostly degraded and undetectable at day 28 (Supplementary Fig. 4). Microscopic observations revealed that a large number of donor MSCs were retained on the heart surface at day 3 after MSC-dressing therapy. Their quantity was reduced by day 28, though still a sizable number of donor MSCs were detected at this time point (Fig. 3a). Only few MSCs migrated into the myocardium. Presence of collagen sheet was identified with its autofluorescence at day 3, whilst it became undetectable at day 28. In contrast, at day 3 after IM injection, donor MSCs formed isolated clusters within the myocardium (Fig. 3b). The size of cell clusters decreased, and only a small number of MSCs were identified by day 28. Semi-quantitative histological assessment of donor cell quantities suggested that the presence of donor MSCs at day 3 was greater using the MSC-dressing technique compared to IM injection (Fig. 3c). The frequency of cleaved caspase-3-positive apoptotic donor MSCs were reduced using the MSC-dressing technique (Fig. 3d), while proliferation of transplanted MSCs was rarely detected in either group as assessed by Ki67 immunohistostaining (Fig. 3e). These suggest that improved maintenance (attenuated cell death), but not increased proliferation, contributed to the increased donor cell quantities using the MSC-dressing technique.

### 3.4 Augmented myocardial tissue repair by MSC-dressing therapy

**Fig. 4:**
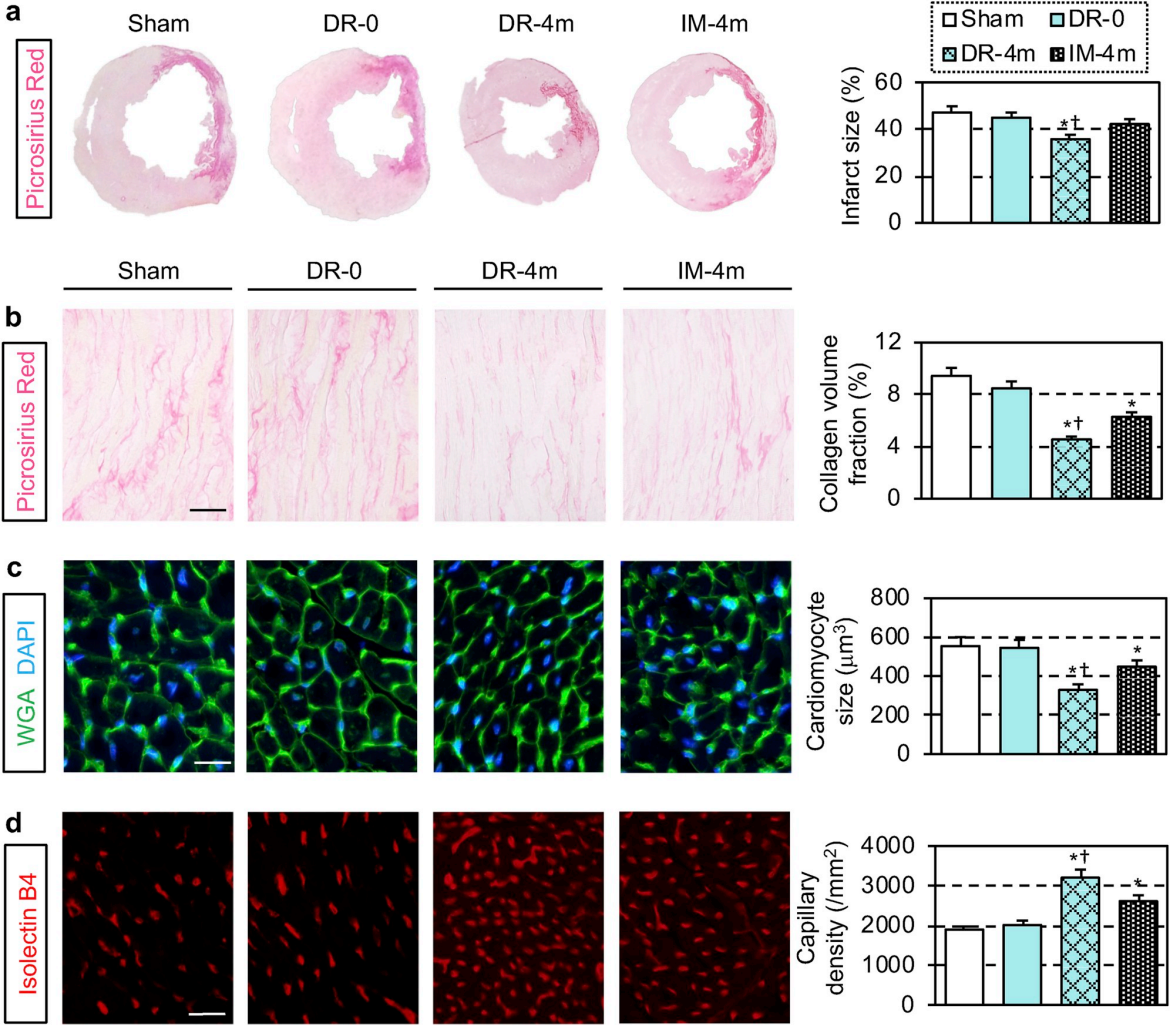
Enhanced repair of the damaged myocardium by MSC-dressing therapy. Heart samples at 4 weeks after transplantation of 4** × **10^6^ rat amnion-derived MSCs (either by MSC-dressing technique or IM injection; DR-4m and IM-4m groups, respectively) in a rat ischemic heart failure model were collected and analyzed by histological assessments to elucidate myocardial repair post MSC-based therapy. The DR-0 group received epicardial transplantation of fibrin sealant film only, while the Sham group received no treatment. (**a**) Representative images of picrosirius red staining of the heart. (**b**–**d**) Collagen volume fraction (**b**), cardiomyocyte hypertrophy (**c**) and capillary density (**d**) in the peri-infarct viable myocardium measured using heart samples stained with/for picrosirius red, wheat germ agglutinin and isolectin B4, respectively. n = 5 in each group, *p < 0.05 vs. Sham and DR-0 groups, ^†^p < 0.05 vs. IM-4m group. One-way ANOVA. Scale bars = 100 μm (b, d) and 25 μm (c). (For interpretation of the references to color in this figure legend, the reader is referred to the Web version of this article.)

Above observed improvement of donor MSC engraftment using the dressing technique was correlated with augmented histological myocardial tissue repair. In addition to the reduced infarct size (Fig. 4a), attenuated pathological interstitial fibrosis, reduced cardiomyocyte hypertrophy and improved microvascular formation were detected in the peri-infarct viable myocardium (Fig. 4b–d) after the MSC-dressing technique and IM injection, compared to the sham treatment group. Of note, the MSC-dressing technique exhibited more extensive effects to increase neo-vascular formation and attenuate pathological fibrosis and cardiomyocyte hypertrophy compared to IM injection of the same number of MSCs. These reparative effects in the host damaged myocardium corresponded to the improvement of cardiac function by MSC-dressing therapy. The placement of a film only (DR-0 group) did not induce such myocardial repair.

### 3.5 Amplified upregulation of tissue repair-related genes by MSC-dressing therapy

**Fig. 5:**
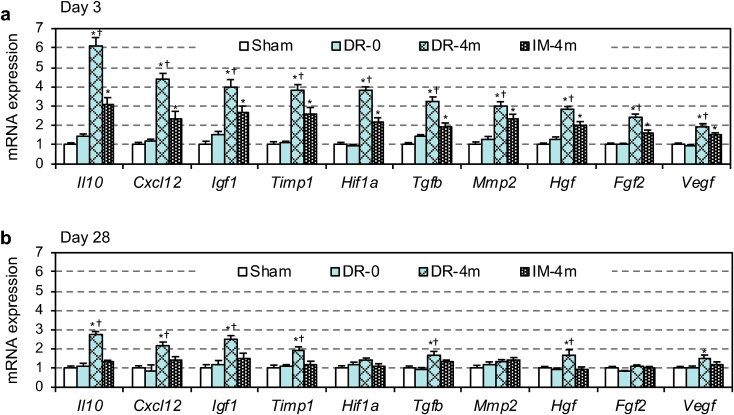
Upregulated tissue repair-related genes by MSC-dressing therapy. Heart samples after transplantation of 4** × **10^6^ rat amnion-derived MSCs (either by MSC-dressing technique or IM injection; DR-4m and IM-4m groups, respectively) in a rat post-MI ischemic heart failure model were collected and assessed for myocardial expression of reparative genes by Real time RT-PCR at day 3 (**a**) and day 28 (**b**) post-treatment. The DR-0 group received transplantation of fibrin sealant film only, while the Sham group received no treatment. Expression in the Sham group was arbitrarily defined as 1.0. n = 5 in each group, *p < 0.05 vs. Sham and DR-0 groups,^*†*^*p < 0.05 vs. IM-4m group. One-way* ANOVA.

Real time RT-PCR elucidated that the MSC-dressing technique facilitated myocardial upregulation of a range of genes related to cardiac repair, including *Il10, Cxcl12, Igf1, Hif1a, Tgfb, Hgf, Fgf2* and *Veg f* [3,36,37]*,* at day 3 post-transplantation compared to both IM injection and sham treatment groups (Fig. 5a). This upregulation corresponded with the histological findings of increased neo-vascular formation and/or reduced fibrosis observed in Section 3.4 (Fig. 4). The majority of these genes were persistently upregulated at day 28 (Fig. 5b). IM injection also induced upregulation of these genes compared to the sham control. However, such upregulation was largely diminished by day 28. The placement of the film only did not induce upregulation of these tissue-repair genes, consistent with the observation that this group failed to induce histological repair of the damaged myocardium.

### 3.6 Long-term therapeutic effects of MSC-dressing therapy in a rat heart failure model

**Fig. 6:**
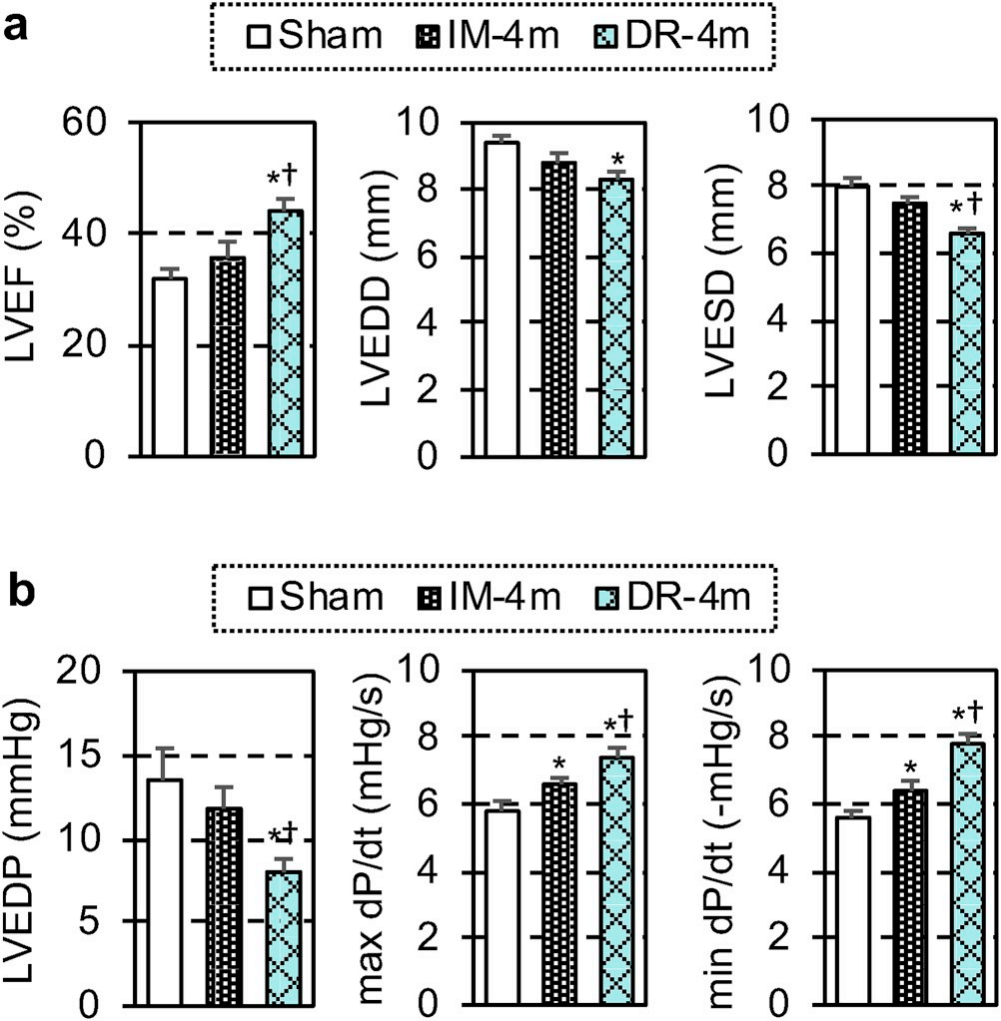
Long-term effect of MSC-dressing therapy in a rat heart failure model. Four weeks after left coronary artery ligation, rats received MSC-dressing therapy (containing 4** × **10^6^ rat amnion-derived MSCs; DR-4m group), IM injection of 4** × **10^6^ rat amnion-derived MSCs (IM-4m group), or no treatment (Sham group). At 90 days after these treatments, cardiac function and structure were measured by using echocardiography (**a**) and cardiac catheterization (**b**). LVEF, left ventricular ejection fraction; LVEDD, left ventricular end-diastolic dimension; LVESD, left ventricular end-systolic dimension; LVEDP, left ventricular end-diastolic pressure, n = 9–10 in each group; *p < 0.05 vs. Sham group, ^†^p < 0.05 vs. IM-4m group. One-way ANOVA.

One of the important questions regarding MSC-based therapy is whether the therapeutic effects will last long. Therefore, we investigated a long-term benefit induced by the MSC-dressing therapy using the same rat ischemic cardiomyopathy model as in Section 3.2. At 90 days post-treatment, echocardiography demonstrated that the MSC-dressing technique persistently improved cardiac function and reduced left ventricular dilatation compared to both sham control and IM injection groups (Fig. 6a). By contrast, improvement of cardiac function by IM injection was not statistically significant compared to the control at this later phase. In addition, catheterization showed both systolic and diastolic cardiac performance remained to be improved by the MSC-dressing therapy and IM injection compared to the control, but with the effect being more extensive using the dressing therapy (Fig. 6b).

### 3.7 Quantitative assessments of donor cell dynamics after MSC-dressing therapy

**Fig. 7:**
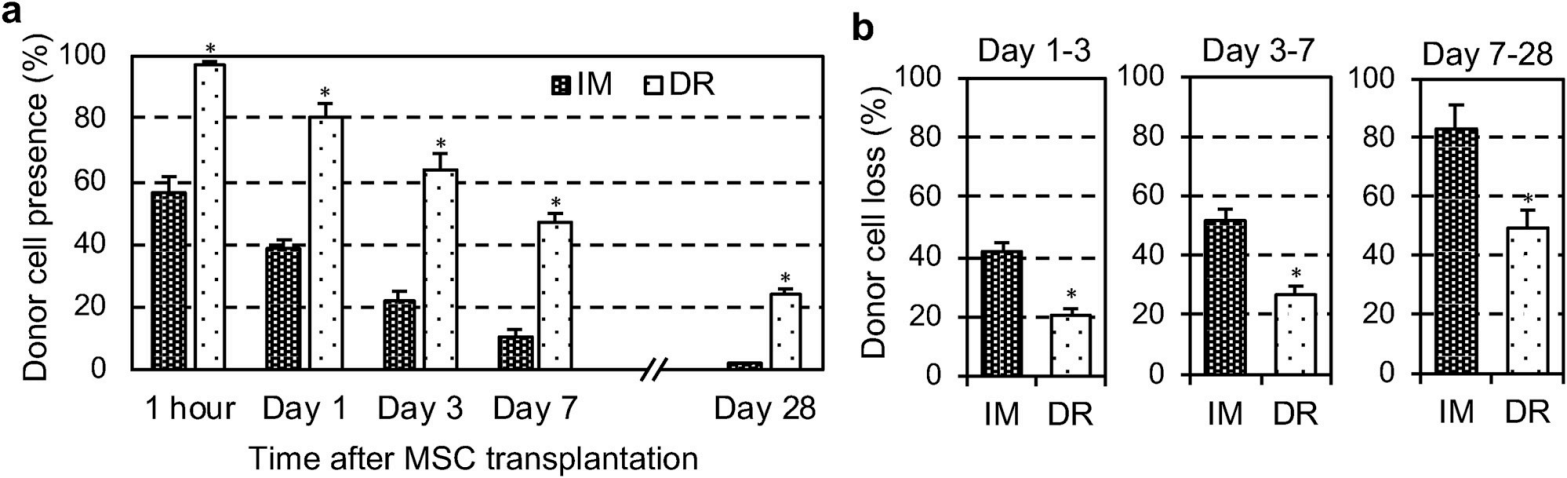
Quantitative assessment of donor cell engraftment after MSC-dressing therapy. (**a**) After transplantation of 4 × 10^6^ male rat bone marrow-derived MSCs into the female rat heart with MI by either the dressing technique (DR group) or intramyocardial injection (IM group), retention/survival of donor MSCs was assessed using quantitative PCR for the male-specific *Sry* gene. Donor cell presence (Y-axis) shows the ratio of the number of existing donor cells to the number of total transplanted cells. n = 4-5; *p < 0.05 vs. IM group, Repeated measures ANOVA. (**b**) Percent loss of donor MSCs after MSC transplantation during the selected periods in the DR and IM groups was calculated from Fig. 7a data. Donor cell loss (Y-axis) means the ratio of the number of disappeared donor cells during the period to the number of existing donor cells at the beginning of the period. n = 4-5; *p < 0.05, Student's t-test.

Section 3.3 demonstrated that the MSC-dressing technique improved donor cell engraftment in the heart; however, this was from semi-quantitative immunohistolabeling-based assessments. As the improved donor cell engraftment is an important mechanism by which MSC-dressing therapy augments the therapeutic efficacy of MSC-based therapy, we further investigated this aspect in a quantitative manner. The most frequently-used, reliable model to this aim is transplantation of male derived MSCs into the female heart, which enabled quantitative assessment of donor cell retention and survival by detecting the male specific gene in the female heart [[13], [14], [15], [16],^31^]. However, unfortunately, amnion-derived MSCs are not suitable for this model, and thereby we alternatively used male rat bone marrow-derived MSCs (see Supplementary Fig. 5 for their characters) as the donor cells to enable this sex-mismatch model. The results obtained revealed that the initial retention of donor MSCs at 1 h post-transplantation was markedly increased to almost 100% using the MSC-dressing technique compared to IM injection of MSC suspension (Fig. 7a). This suggested that the leakage and/or death of donor cells shortly after transplantation, which are major causes of poor donor cell engraftment after IM injection and intracoronary injection [[6], [7], [8]], were effectively prevented by the dressing technique. With time, donor cell presence decreased in both groups; however, throughout the time course studied, the dressing technique exhibited greater donor MSC presence compared to IM injection. In addition, calculated ratios of donor cell-loss between day 1 and day 3, between day 3 and day 7 and between day 7 and day 28 post-transplantation were all attenuated by the dressing technique (Fig. 7b). This suggested that MSCs transplanted by the dressing technique were provided with a more suitable environment and better maintained compared to those transplanted by IM injection.

### 3.8 Fibrin-induced upregulation of tissue repair-related genes in incorporated MSCs

**Fig. 8:**
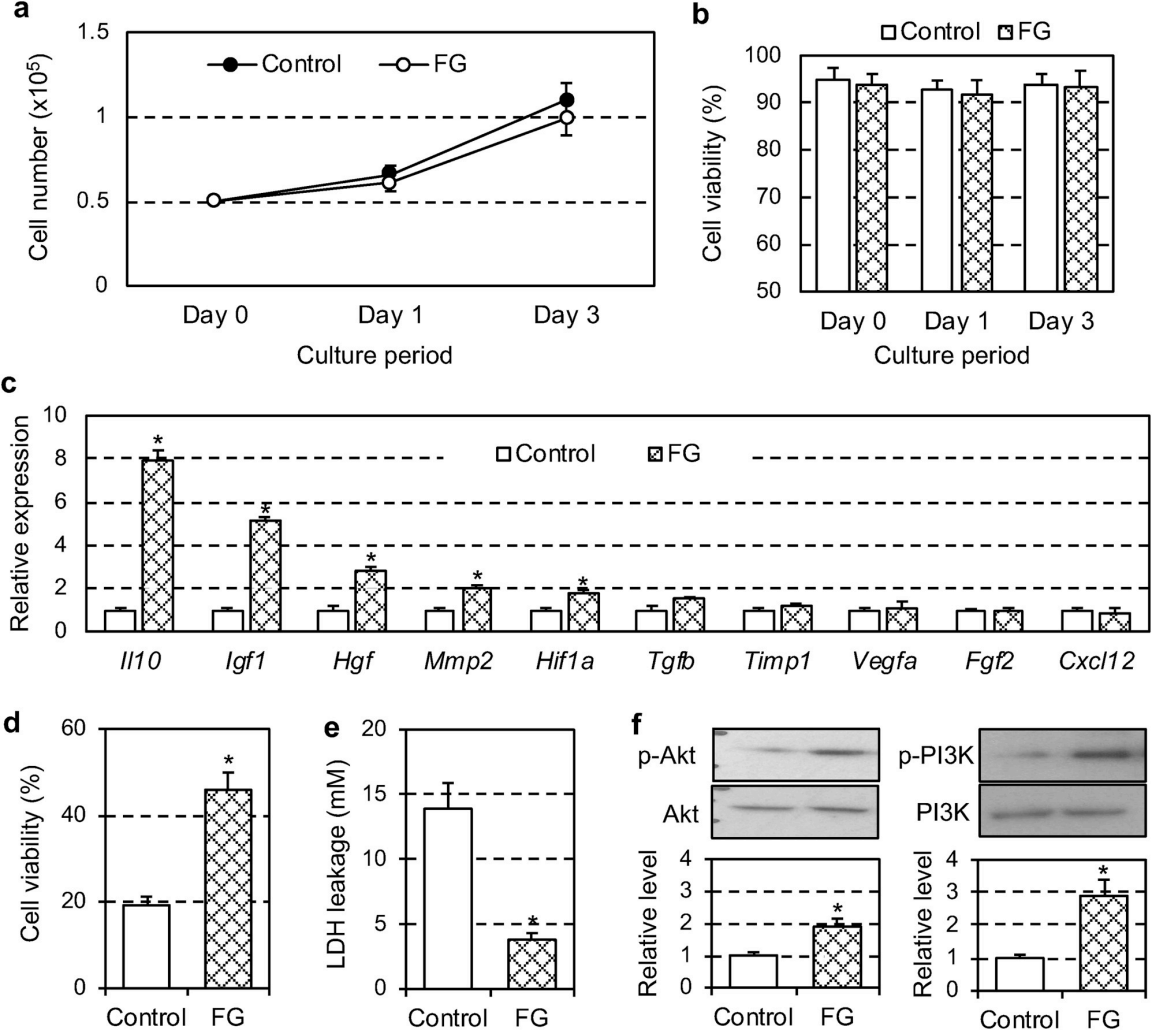
Fibrin-induced reparative gene expression and cell survival signal in donor MSCs. Rat amnion-derived MSCs were cultured in fibrin glue (FG group) or standard growth medium (Control group) *in vitro*. n = 6 in each group, *p < 0.05. (**a**) Changes in cell numbers after plating 0.5 × 10^5^ MSCs were monitored at chosen time points. *Repeated measures ANOVA (not significant).* (**b**) Viability of cultured MSCs were measured at chosen time points during cultivation. *Repeated measures ANOVA (not significant).* (**c**) Gene expression of MSCs at Day 3 was assessed by real time RT-PCR. Expression in the Control group was arbitrarily defined as 1.0. *Student's t-test.* (**d, e**) Viability of MSCs (**d**) and LDH leakage into the medium (**e**) was assessed after administration of H_2_O_2_ to the MSC culture. *Student's t-test.* (**f**) Expression and activation of Akt and PI3K in MSCs were measured by Western blot. The proportion of the phosphorylated protein to the total protein was normalized to the Control group. *Student's t-test.*

We additionally hypothesized that enclosure in a fibrin gel (a major component of the MSC-dressing) would modulate the cellular properties of donor MSCs, which could in turn influence the potential of MSCs for myocardial repair. Our *in vitro* studies demonstrated that, while the growth and viability of fibrin-cultured MSCs were as high as those in standard growth medium [[13], [14], [15], [16]] (Fig. 8a and b), culture in a fibrin glue upregulated a range of tissue repair-related genes in MSCs (Fig. 8c). The spectrum of the upregulated genes, including *Il10, Igf1, Hgf* and *Hif1a,* was overlapped with those upregulated after MSC-dressing technique *in vivo* (Fig. 5a). These results suggest that not only the increased number of surviving donor MSCs but also enhanced reparative functions of surviving donor cells could contribute to the augmented therapeutic effect elicited by the MSC-dressing technique.

### 3.9 Activated cell survival signals in MSCs by fibrin

We next investigated whether and how fibrin would protect incorporated MSCs from external insult using an *in vitro* model. In response to H_2_O_2_ challenge, ordinarily-cultured MSCs showed extensive cell death with increased LDH leakage, whilst such MSC death was attenuated when cultured in a fibrin gel (Fig. 8d and e). Of note, we found that this increased survival in fibrin-integrating MSCs was associated with a robust activation of cell survival pathway, including Akt/PI3K [38] (Fig. 8f). This fibrin-mediated activation of the intrinsic self-protection system is likely to contribute to the enhanced survival of transplanted MSCs using the dressing technique *in vivo*.

## 4 Discussion

We here described a novel biomedical engineering method, the MSC-dressing technique, which enhances the efficacy of MSC-based therapy for heart failure. Production, handling and placement of the MSC-dressing were simple and easy. Upon placement, the MSC-dressing promptly and persistently adhered to the epicardial surface of beating hearts without sutures or additional glues. While the collagen and fibrin components were gradually degraded and absorbed *in vivo*, a sizable number of transplanted MSCs retained on the heart surface. Of note, this technique attained augmentation of both initial retention and subsequent survival of donor MSCs compared to the widely-used IM injection method. *In vitro* studies revealed that the fibrin gel enhanced the quality of integrated MSCs through magnifying their tissue-repair function and also activating the Akt/PI3K-involved self-protection system. These multiple benefits collectively resulted in the enhanced therapeutic effects in a clinically relevant rat model of post-MI ischemic heart failure. The therapeutic effects of the MSC-dressing therapy were proportional to the number of MSCs transplanted, assuring the functional contribution of MSCs. These MSC-dressing therapy-mediated benefits were underpinned by enhanced myocardial tissue repair, including increased neo-vascularization, attenuated pathological fibrosis and reduced cardiomyocyte hypertrophy in the viable peri-infarct myocardium. These effects corresponded to augmented myocardial upregulation of a group of tissue repair-related genes. In addition to these advantages in the enhanced therapeutic efficacy, the dressing technique offers the ease of practical application. After delivery of a film and an MSC product from a hub center/company, the MSC-dressing can be instantly produced at the point of the treatment. As such, this cell therapy can be performed at a hospital even that does not have a cell processing center. These features will allow the MSC-dressing therapy to become a widely adopted standard treatment for heart failure.

The MSC-dressing technique improved not only the initial retention of transplanted MSCs but also the subsequent maintenance (survival) of retained MSCs. Increased initial retention was achieved due to the adhesive nature of fibrin, which enabled the MSC-dressing to firmly adhere to the heart, while securely retaining donor MSCs within. As such, leakage of donor cells early after transplantation, which is a major cause of poor donor cell engraftment using the current methods [7,8], were largely prevented. Since TachoSil^Ⓡ^ is a prefabricated product, the fibrin concentration or crosslinking strength can be changed only marginally between conditions i.e. types and volumes of solution/serum added [[39], [40], [41]]. Therefore, it is considered that the adhesion strength of MSC-dressing is around 50-70 hPa, comparable to that of TachoSil^Ⓡ^ [[39], [40], [41]]. In addition, maintenance of donor cells was improved by the MSC-dressing technique with apoptosis of donor MSCs being attenuated. We speculate that this improved maintenance is underpinned by multiple mechanisms. Firstly, both collagen sheet and fibrin gel composing of the MSC-dressing would offer protection of donor MSCs from physical (i.e. abrasion by surrounding tissues) and chemical hazards (i.e. free radicals) by acting as a barrier [42]. Secondly, fibrin is known to provide a suitable three-dimensional scaffold to MSCs [16], maintaining high viability and functional potential of the cells. Thirdly, our results uncovered that fibrin-based culture activated the Akt/PI3K cell-survival signal in MSCs and thus strengthened their intrinsic self-protection ability against external stress.

As regards the degradation time of MSC-dressing, we confirmed that MSC-dressings remained undegraded on the heart surface at day 3 post-transplantation, but largely degraded by day 28. This degradation course appears to be quicker compared to TaschoSil degradation reported in previous literature (>12 weeks) [27,28]. Degradation time of TachoSil or MSC-dressings will depend on the localization, in which these are placed. Particularly, MSC-dressings placed on the heart surface (in the pericardial space) are likely to suffer continuous mechanical abrasion and stress from the surrounding tissue accompanied by heartbeat, which may hasten degradation of the MSC-dressing. In addition, the presence of MSCs may also accelerate the degradation through their biological effect.

In addition to such increased quantities of donor cell engraftment, our data also suggest that the reparative capability of donor MSCs were amplified by the use of the MSC-dressing technique. Culture in fibrin induced upregulation of *Il10, Igf1, Hgf,* and *Hif1a* in MSCs, all of which are known key players in myocardial repair post-MI [3,36,37]. Kim et al. have previously reported that fibrin-cultured MSCs increased expression of HGF [43], and our study revealed the impact of fibrin on a wider range of tissue repair-related genes. Although the molecular mechanism underpinning the fibrin-mediated upregulation of tissue repair-related genes in MSCs remains uncertain, one potential explanation is that the characteristic components of the fibrin matrix, arginine-glycine-asparagine motifs, may bind to integrin families expressed on the surface of MSCs to mediate their intracellular signal cascades, resulting in amplified expression of reparative genes [[44], [45], [46], [47]]. In addition, fibrin may enhance the MSC-mediated paracrine effect through binding certain kinds of growth factors at heparin-binding domains [48], which can act as a reservoir for sustained-release of the growth factors [42]. Further focused studies are needed to elucidate the mechanism of interaction between fibrin and MSCs. Among upregulated reparative genes in the myocardium *in vivo* (Fig. 5), *Il-10, Igf1, Hif1a, Mmp2* and *Hgf* were upregulated in MSCs when cultured in fibrin *in vitro* (Fig. 8c). On the other hand, remaining genes, including *Cxcl12, Fgf2* and *Vegf,* were upregulated in the myocardium *in vivo*, but not in fibrin-cultured MSCs *in vitro*. We speculate that this inconsistency would cause because myocardial gene expression was the mixture of many cell types including not only donor MSCs but also host heart cells such as cardiomyocytes, fibroblasts and immune cells. It is likely that host heart cells upregulate reparative genes in response to myocardial injury and MSC transplantation.

In the clinical setting, it will be the most effective to apply the MSC-dressing technique to an already exposed heart under thoracotomy and pericardiotomy. Thus, we initially plan to add this therapy to open heart surgery, i.e., coronary artery bypass grafting (CABG) to ischemic cardiomyopathy or left ventricular assist devise (LVAD) implantation for end-stage heart failure. Importantly, the outcome of these current operations remains unsatisfactory [49,50], requiring refined strategies to improve the prognosis of relevant patients. It is estimated that there are more than 4000 patients who suffer ischemic cardiomyopathy and receive CABG every year in the UK (data from http://www.bluebook.scts.org as of January 2019). We expect that an addition of MSC-dressing therapy to these surgical treatments is likely to induce synergistic/additional therapeutic effects as previously reported [51,52]. Enhanced microvascular formation by the MSC-dressing therapy and increased blood flow by CABG will result in augmented improvement of functional myocardial perfusion. Ventricular unloading by LVAD may provide an increased opportunity for the failing myocardium to respond to the reparative paracrine stimuli from transplanted MSCs. In turn, increased myocardial perfusion or reduced environmental stress by unloading may improve donor MSC survival, leading to further improvement of the overall therapeutic outcome. All procedures for the MSC-dressing therapy are so easy that it will add only negligible burden to clinicians.

This study provided pre-clinical evidence of the efficacy of MSC-dressing therapy for ischemic heart failure in rat models. To the end of its clinical application, however, we need to be careful about species differences between the rat and human hearts. Although the MSC-dressing was able to achieve the successful paracrine effect in the thin (∼1 mm) rat ventricular walls, it remains uncertain whether the secretome from epicardially placed MSC-dressings can diffuse sufficiently deep into the much thicker (>1 cm) walls of human hearts. Given the promising results in the previous large animal and clinical studies, in which epicardially placed cardiac patches induced significant therapeutic effects [[19], [20], [21], [22], [23]], this may be an overconcern. Nonetheless, an addition of appropriate large animal studies to solve this uncertainty could strengthen the potential of clinical success of the MSC-dressing therapy. Needless to say, towards the success of MSC-Dressing therapy, it is also essential to establish the protocols to produce and transport a high-quality human MSCs in a reproducible manner.

## 5 Conclusion

This translational study provides pre-clinical proof of concept to support the MSC-dressing therapy for ischemic heart failure. The MSC-dressing therapy was feasible, improved cardiac function, and reduced heart dilatation in a dose-dependent manner in a rat ischemic cardiomyopathy model. Furthermore, these therapeutic effects of MSC-dressing therapy were greater than those of intramyocardial MSC injection, which is a current common method. Increase in LVEF compared to the control group was 13.4% and 5.3% by MSC-dressing therapy and intramyocardial injection, respectively (Fig. 2). This improvement by intramyocardial injection is consistent to that observed in previous clinical trials [53], and of note, MSC-dressing therapy exhibited 2.5-fold greater improvement compared to this. MSC-dressing therapy is also user-friendly, requiring no specialized equipment in the hospital, thus having a great potential to be widely adopted therapy for heart failure. Continuous pre-clinical and clinical development is warranted for the success of this innovative therapy.

## Data Availability

The raw/processed data required to reproduce these findings cannot be shared at this time as the data also forms part of an ongoing study.
