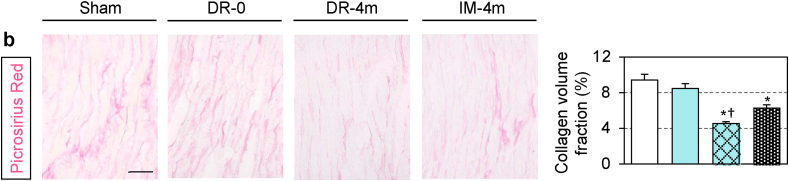# Corrigendum to “On-site fabrication of bi-layered adhesive mesenchymal stromal cell dressings for the treatment of heart failure” [Biomaterials 209 (2019) 41–53]

**DOI:** 10.1016/j.biomaterials.2024.122499

**Published:** 2024-04

**Authors:** Kazuya Kobayashi, Yuki Ichihara, Nobuhiko Sato, Nobuyoshi Umeda, Laura Fields, Masafumi Fukumitsu, Yoshiyuki Tago, Tomoya Ito, Satoshi Kainuma, Mihai Podaru, Fiona Lewis-McDougall, Kenichi Yamahara, Rakesh Uppal, Ken Suzuki

**Affiliations:** a William Harvey Research Institute, Barts and the London School of Medicine, Queen Mary University of London, United Kingdom; b Kaneka Corporation, Osaka, Japan; c Transfusion Medicine and Cellular Therapy, Hyogo College of Medicine, Japan

The authors regret that there was an error in Fig 4b of this paper (DR-0 panel). The corrected version is shown below. This correction does not alter the results, graphs, or conclusions of the study. The authors would like to apologise for any inconvenience caused.

**Figure:**