# Supplementary material for: On-site fabrication of Bi-layered adhesive mesenchymal stromal cell-dressings for the treatment of heart failure
Source: Biomaterials. 2019 Jul;209:41–53. doi: 10.1016/j.biomaterials.2019.04.014 (PMC6527869; doi:10.1016/j.biomaterials.2019.04.014)
Supplement: Multimedia component 1 [file mmc1.pdf]

<u>SUPPLEMENTARY INFORMATION</u>

On-site Fabrication of Bi-layered Adhesive Mesenchymal Stromal Cell-Dressings for the Treatment of Heart Failure

- Supplementary Fig. 1. Characterization of human amnion-derived MSCs
- Supplementary Fig. 2. Optimization of the protocol to produce an MSC-dressing
- Supplementary Fig. 3. Characterization of rat amnion-derived MSCs
- Supplementary Fig. 4. Macroscopic observation of MSC-dressings on the rat heart *in vivo*.
- Supplementary Fig. 5. Characterization of rat bone marrow-derived MSCs
- Supplementary Table 1. Pre-treatment echocardiography data in the rat heart failure model.

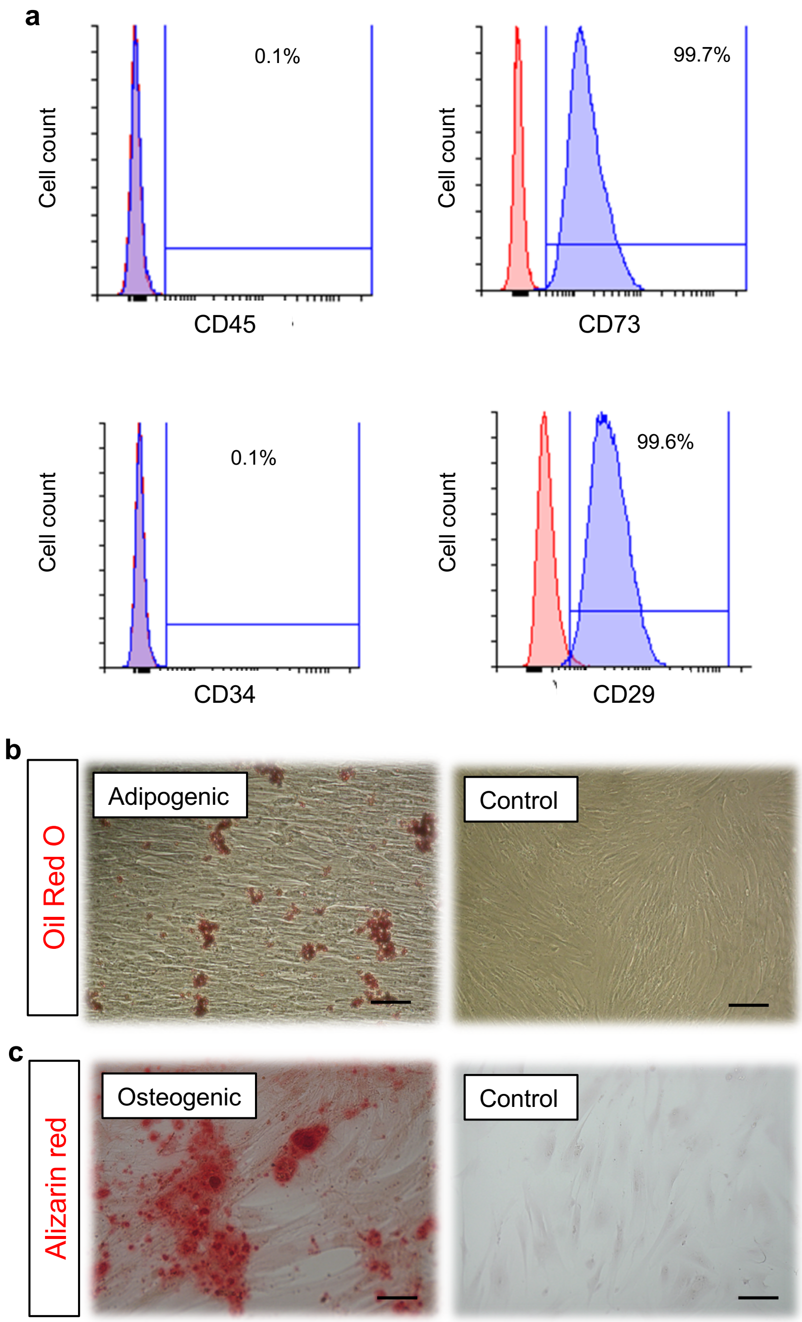

Supplementary Fig. 1. Characterization of human amnion-derived MSCs

(**a**) CD marker expression of human amnion-derived MSCs (passage 4) assessed by flow cytometric analysis. *Red; IgG control, Purple; CD45/CD73/CD34/CD29 staining.*

(**b**, **c**) Differentiation of human amnion-derived MSCs under adipogenic stimulation (**b**; Oil Red-O staining) and osteogenic stimulation (**c**; Alizarin red staining). *Control; cultured in usual medium; Scale bars=50 µm (**b**) and 15 µm (**c**).*

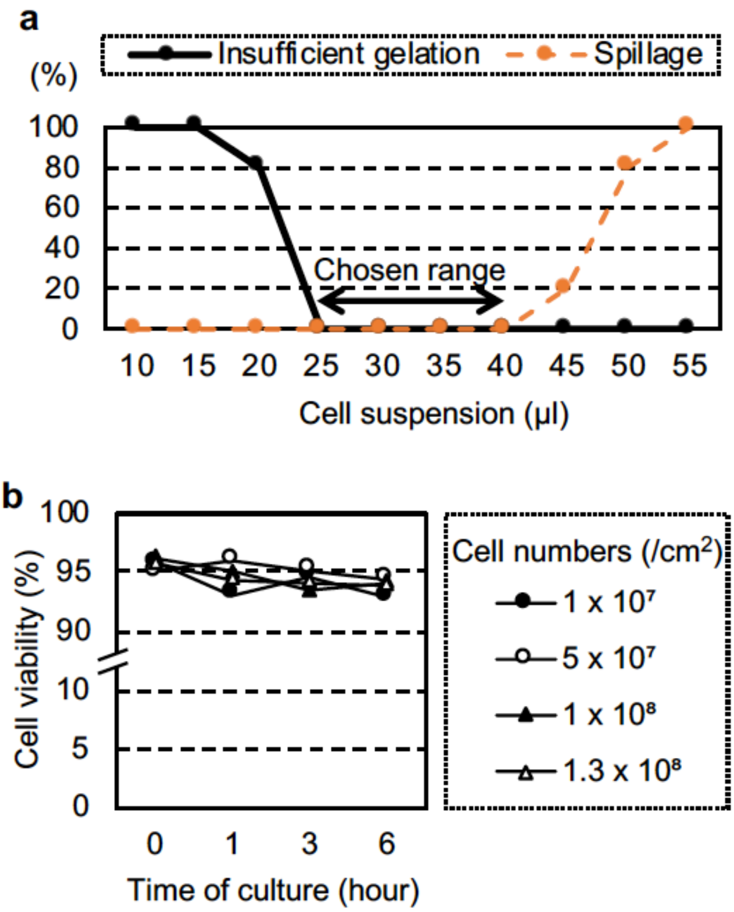

Supplementary Fig. 2. Optimization of the protocol to produce an MSC-dressing

(**a**) *Optimization of the MSC suspension volume to produce an MSC-dressing.* Human amnion-derived MSC suspension (10-55 µl) was spread onto the fibrinogen/thrombin-coated side of 1 cm^2 fibrin sealant film. Homogenous fibrin production and spillage of MSC-suspension when inverted were visually assessed. *n=5 samples in each condition.*

(**b**) *Optimization of the MSC-density to produce an MSC-dressing.* Human amnion-derived MSC suspension (30 µl including 1×10^7 - 1.3×10^8 cell/ml) were loaded onto the fibrinogen/thrombin-coated side of 1 cm^2 sealant film, and the viability of MSCs was assessed. *n=5 samples examined for each condition.*

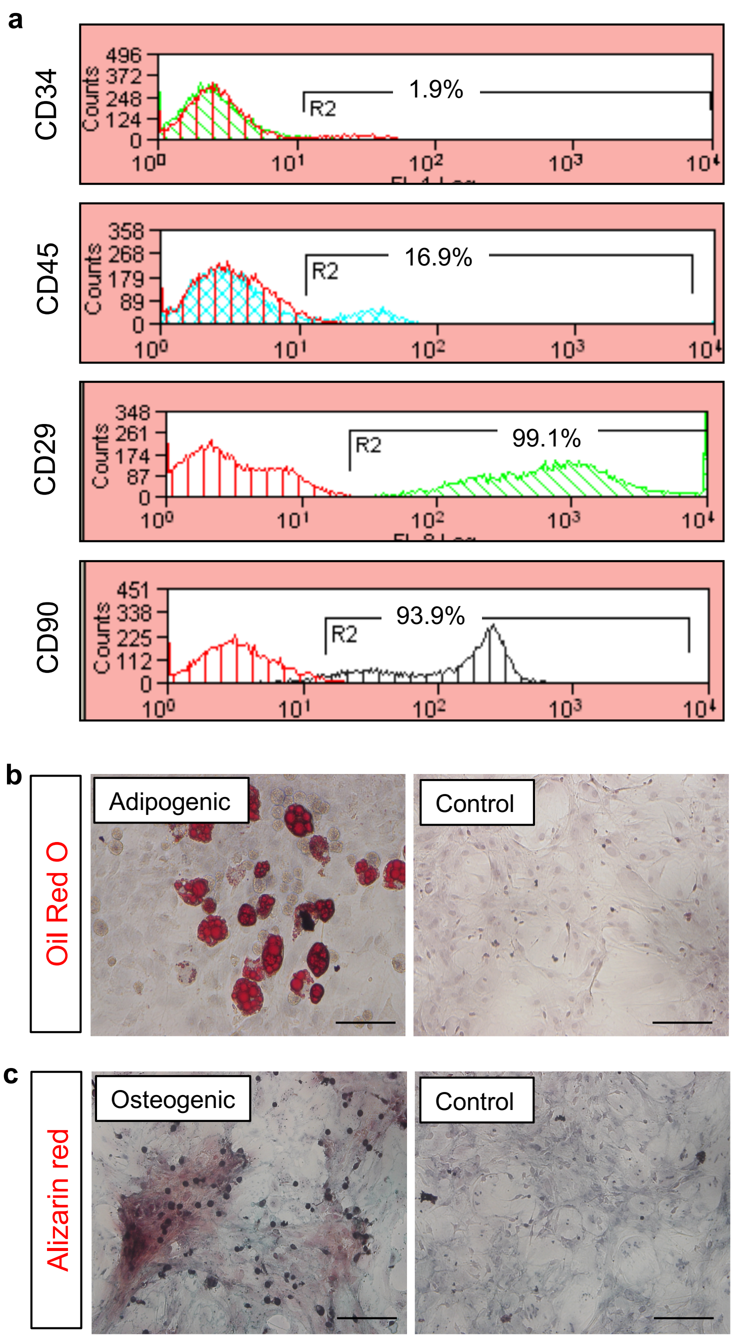

Supplementary Fig. 3. Characterization of rat amnion-derived MSCs

(**a**) CD marker expression of rat amnion-derived MSCs (passage 4) assessed by flow cytometric analysis. *Red in each panel; IgG control.*

(**b**, **c**) Differentiation of rat amnion-derived MSCs under adipogenic stimulation (**b**; Oil Red-O staining) and osteogenic stimulation (**c**; Alizarin red staining). *Control; cultured in usual medium; Scale bars=100 µm.*

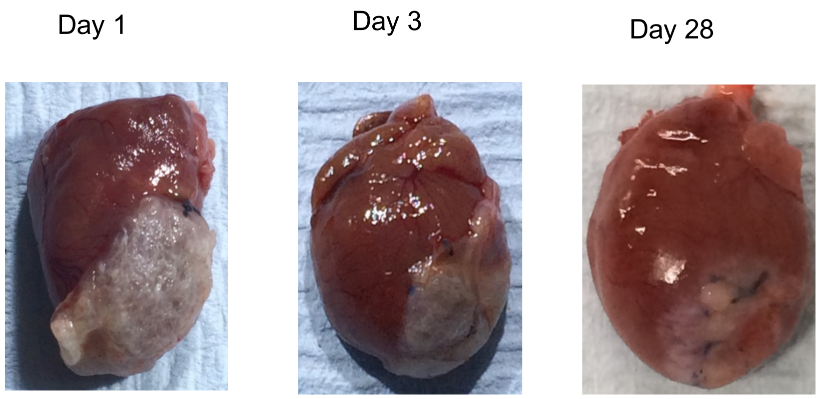

Supplementary Fig. 4. Macroscopic observation of MSC-dressings on the rat heart in vivo

Four weeks after left coronary artery ligation in rats, 1 cm^2 MSC-dressing containing 4×10^6 rat amnion-derived MSCs were placed onto the rat heart surface. At chosen time points after the treatment, the hearts were removed and observed macroscopically.

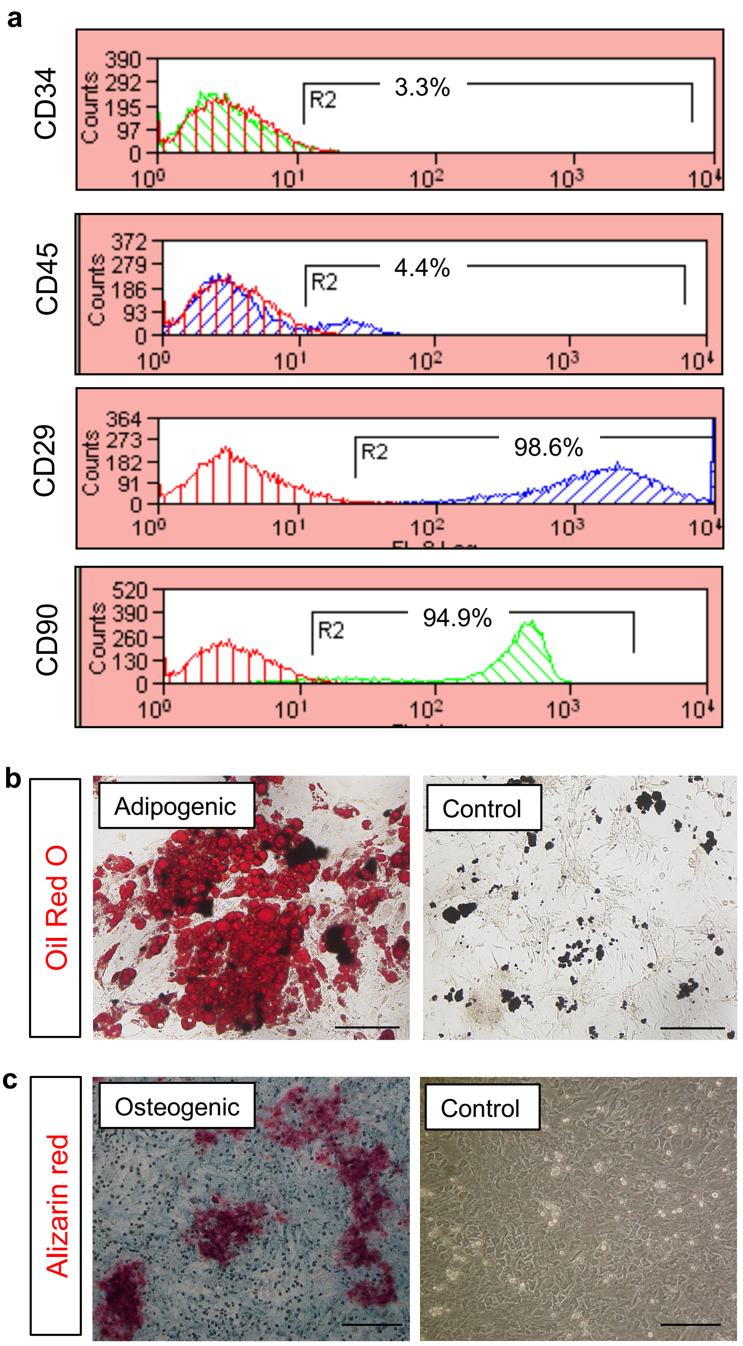

Supplementary Fig. 5. Characterization of rat bone marrow-derived MSCs

(**a**) CD marker expression of rat bone marrow-derived MSCs (passage 4 or 5) assessed by flow cytometric analysis. *Red in each panel; IgG control.*

(**b**, **c**) Differentiation of rat bone marrow-derived MSCs to adipocyte under adipogenic stimulation (**b**; Oil Red-O staining) and osteocytes under osteogenic stimulation (**c**; Alizarin red staining). *Control; cultured in usual medium; Scale bars=100 μm.*

Pre-treatment echocardiography data (4 weeks after MI)

Group	n	HR (bpm)	LVEF (%)	LVESD (mm)	LVEDD (mm)
**Sham**	10	379.8±7.8	36.9±2.4	6.81±0.48	8.44±0.42
**DR-0**	10	389.0±6.7	35.8±1.8	7.02±0.29	8.50±0.39
**DR-1m**	10	375.7±8.2	37.1±2.2	6.84±0.24	8.48±0.34
**DR-2m**	10	377.3±8.8	36.8±1.7	6.89±0.30	8.46±0.35
**DR-4m**	10	378.9±9.1	36.7±1.5	6.90±0.33	8.39±0.38
**IM-4m**	10	383.6±7.9	35.9±1.9	6.98±0.27	8.42±0.35

Supplementary Table 1. Pre-treatment echocardiography data in the rat heart failure model

Cardiac function of rats was measured by using echocardiography before treatment (at 4 weeks after left coronary artery ligation; post-MI ischemic cardiomyopathy model). No significant difference was detected in any indicators among any groups (One-way ANOVA). *LVEF, left ventricular ejection fraction; LVEDD, left ventricular end-diastolic dimension; LVESD, left ventricular end-systolic dimension; LVEDP, left ventricular end-diastolic pressure.*
